# Comparison of Different Encapsulation Techniques in Exosomes Obtained from Goat and Bovine Milk

**DOI:** 10.1021/acsomega.5c09524

**Published:** 2025-12-16

**Authors:** Mariana Cavalcante Theophilo Gaspar de Oliveira, Helen Paula Silva Costa, Daniel Teixeira, Daniele Oliveira Bezerra Sousa, Carla Renata Figueiredo Gadelha, Maria Izabel Florindo Guedes, Maurício Fraga van Tilburg

**Affiliations:** † Biotechnology and Molecular Biology Laboratory, 162380 State University of Ceará, 1700, Dr. Silas Munguba Avenue, Fortaleza, Ceará CEP: 60714-903, Brazil; ‡ Health Science Center, 28128 University of Fortaleza, 1321, Washington Soares Avenue, Fortaleza, Ceará CEP: 60811-905, Brazil; § Department of Biochemistry and Molecular Biology, 28121 Federal University of Ceará, 2977, Mister Hull Avenue, 907 Block, Fortaleza, Ceará CEP 60440-900, Brazil; ∥ 676567 Federal University of Ceará − Pici Campus, Department of Animal Science, 2977, Mister Hull Avenue, 808 Block, Fortaleza, Ceará, CEP: 60440-554, Brazil; ⊥ Biotechnology Post-Graduation Program, State University of Ceará, 1700, Dr. Silas Munguba Avenue, Fortaleza, CE CEP: 60714-903, Brazil

## Abstract

Exosomes are the smallest extracellular vesicles and can transport several molecules between cells. Mammalian milk is a significant source of exosomes. The aim of this study was to isolate exosomes from goat milk (GM) and cow milk (CM) to compare their properties and their capacities for loading with bioactive compounds. Exosomes were purified by differential ultracentrifugation and morphologically characterized by scanning electron microscopy (SEM) and dynamic light scattering (DLS). *Moringa oleifera*—chitin-binding protein *(Mo*-CBP_3_) conjugated with FITC was charged into the vesicles by four different active methods: (1) electroporation, (2) sonication, (3) freeze–thaw cycles, and (4) incubation at room temperature in the presence of saponin. DLS analysis showed no significant difference between the diameters of the two exosome species. All methods of protein charging were effective. The *in vitro* cell assay also confirmed successful endocytosis of exosomes from both species by HepG2 cells, particularly those loaded using the freeze–thaw cycle and sonication methods. Goat milk is a viable alternative for encapsulating and delivering bioactive molecules.

## Graphical abstract

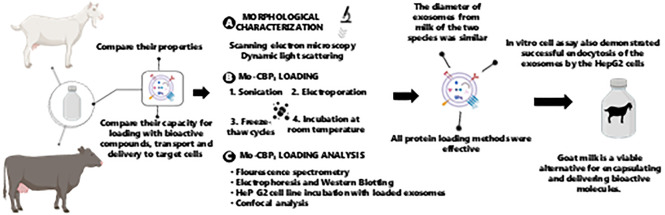

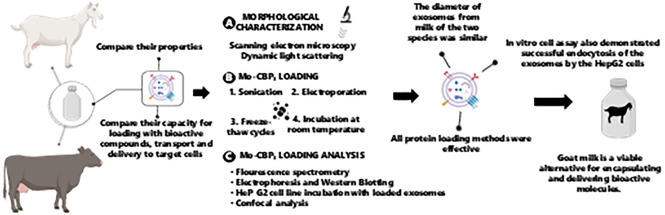

## 1 Introduction

Extracellular vesicles (EVs) are natural transporters directly involved in mediating the physiological functions of cells. Their increasing scientific emphasis is due to their high efficiency in delivering molecules to target cells.^1^ They originate from organelles known as endosomes and are formed from an invagination or budding processes of the cell membrane, which is why they are often considered to be copies of their original cells.^2^ The generation of EVs involves complex molecular networks that function cooperatively or independently to mediate membrane invagination and budding, and the selective sorting of cargo molecules. These processes are tightly regulated by post-translational modifications of proteins and lipids, and possibly by glucose availability.^3^

Exosomes are the smallest extracellular vesicles, with a diameter of about 30–200 nm. Their main function is to transport proteins, nucleic acids, and lipids between cells. Their extremely small size makes them capable of crossing biological barriers and release therapeutic molecules, making them highly promising as potential biopharmaceuticals.^4^ The complexity and heterogeneity of their functions, both under normal and pathological conditions, highlight exosomes as a significant research focus for their application in the therapy and diagnosis of various diseases.^5^

However, their application in clinical research assays as drug delivery systems requires the development of commercially viable and biocompatible sources. Milk exosomes are an important alternative option for exosome isolation, compared to cells, tissues, body fluids, and microorganisms, due to their low cost, convenient extraction methods, biocompatibility, lack of systemic toxicity, and minimal adverse immune and inflammatory responses.^6^ In this context, goat milk emerges as a possible origin in comparison to coẃs milk in the Northeast of Brazil, especially in Ceará, which has one of the largest goat herds in the country, with about 1.13 million animals. This species can easily adapt to the semiarid environment that characterizes about 92% of the state. The herd requires low management costs, which makes the large-scale production of goat products even more profitable.

Although exosomes hold great promise as biopharmaceuticals, their therapeutic application faces challenges, which motivates the pursuit of engineering processes aimed at enhancing their loading capacity with chemicals, drugs, bioactive compounds, proteins, and other cargos of interest.^7^ The main exosome engineering methods include direct or postisolation approaches (on the nanoparticle itself)^8^ or indirect interventions, which involve encapsulating cargos from the parental cells.^9^

Generally, the encapsulation of therapeutic molecules consists of passive (coincubation) or active (membrane permeability enhancement) methods,^10^ and its effectiveness depends on the hydrophobic or hydrophilic nature of the charge.^11^ Small hydrophobic molecules, such as curcumin^12^ or the established chemotherapeutic doxorubicin^13^ are often loaded by simple incubation at room temperature due to their high capacity of interaction with the exosomal membrane. However, hydrophilic or high molecular weight charges, such as proteins or nucleic acids, is difficult to load into exosomes in this way. Therefore, various drug encapsulation methods have been developed to increase the loading capacity regardless of the chemical or physical properties of the cargos.^10^ Thus, in the present study, the challenge of encapsulating a hydrophilic protein *(Mo*-CBP_3_) with well-established chemical and morphological structures was chosen.

Moreover, previous evidence has demonstrated a greater drug-loading capacity of goat milk exosomes for chemotherapeutics (Doxorubicin) compared to exosomes from other species (buffalo and cow). The authors also observed a higher capacity to inhibit tumor cell viability, as well as higher apoptosis induction from GM, compared to BM, CM, and even the drug in its free form. Finally, it was found that unloaded goat milk exosomes could significantly reduce cell viability, suggesting an antiproliferative potential of the isolated nanoparticle itself.^14^

Therefore, this study aimed to evaluate and compare the loading efficiency of four different active techniques in goat and bovine milk-purified exosomes, using a hydrophilic protein (*Mo*-CBP_3_) as a model of bioactive charge, and then analyze the *in vitro* delivery capacity of these systems by incubating them with human hepatocarcinoma cells (HepG2).

## 2 Results and Discussion

Cow milk emerged as a superior source for the isolation of exosomes compared with other alternatives previously studied. Factors such as low cost, easy access, wide distribution, high bioavailability, and no toxicity when orally administered have made cow milk exosomes the focus of interest for researchers in the past few years.^15^

In terms of scalability, milk represents a highly efficient source of exosomal isolation. Commercial semiskimmed cow milk provides substantial exosome yields, with approximately 200 mg of protein per liter when isolated using protocols comparable to ours, involving differential ultracentrifugation followed by filtration.^16^ Remarkably, even simpler procedures employing only differential ultracentrifugation, as used in this study, have achieved higher yields of approximately 335 ± 48 mg per liter (Table 1). This represents over a 200-fold increase compared with other exosome sources, such as cell culture supernatants (0.5–2.0 mg·L^–1^). Therefore, for drug delivery applications, the isolation of exosomes from milk remains particularly advantageous due to its scalability and recovery efficiency.^15^

**Table 1:** Protein Yield Comparison between Isolation Methods and Sources^a^

Study/Reference	Milk Source	Isolation Method	Additional Steps	Protein Yield (mg·L^–1^)	Notes/Comments
Munagala et al., 2016 ^15^	Cow (midlactation period)	Differential ultracentrifugation	None	335 ± 48	Used for drug loading; same method as present study
Arntz et al., 2015 ^16^	Cow (semiskimmed, UHT)	Differential ultracentrifugation +0.2 μm syringe Filtration	None	200	Commercial milk (same sample as present study); low purity and yield
Santos-Coquillat et al., 2021 ^17^	Goat (UHT)	Differential ultracentrifugation + SEC	Enrichment step	3.4–8.5	Higher purity; low yield
Present study (2025)	Cow (UHT)/goat (UHT)	Differential ultracentrifugation	None	389.6 (Cow) 475.3 (goat)	Practical balance between yield and scalability

a Comparison of the protein yield among studies using different isolation strategies and milk sources. Protein recovery is expressed as mg·L^–1^ of starting material, highlighting the influence of isolation complexity and matrix processing on yield efficiency.

In contrast, Santos-Coquillat et al. employed a more complex isolation protocol, combining differential ultracentrifugation with size-exclusion chromatography (SEC) for exosomes derived from UHT goat milk, which markedly reduced the overall protein recovery. After normalization to the initial sample volume (60 mL), their yields corresponded to only 3.4–8.5 mg of protein per liter of milk, nearly 2 orders of magnitude lower than those obtained with ultracentrifugation alone (Table 1).^17^

In this study, the use of a simple and efficient differential ultracentrifugation protocol enabled the effective recovery of milk-derived exosomes. After normalization to an initial milk volume of 1 L, the exosomal protein yield reached approximately 389.6 mg/L for bovine and 475.3 mg/L for caprine samples, exceeding previously reported values for milk-derived vesicles. Notably, these yields surpassed those obtained by Munagala et al.,^15^ who employed the same isolation protocol (Table 1).

In contrast, the particle concentration data obtained by NTA revealed a different trend. Studies combining traditional isolation approaches, such as differential ultracentrifugation, with enrichment protocols like size-exclusion chromatography (SEC) tend to achieve higher nanoparticle recoveries, reaching approximately 1.4 × 10^11^ particles/mL.^18^ Conversely, simpler methodologies employing ultracentrifugation coupled only with a 0.2 μm syringe filter reported considerably lower concentrations, around 2.5 × 10^7^ particles/mL (Table 2).^16^ In our study, the average particle concentrations reached 9.3 × 10^9^ particles/mL for bovine milk and 7.0 × 10^9^ particles/mL for caprine milk, values substantially higher than those reported for minimally processed samples and aligned with the lower range reported for enriched preparations, demonstrating the reproducibility of our method.

**Table 2:** Particle Yield by NTA and DLS Analyses^a^

Study/Reference	Milk Source	Isolation Method	Technique	Yield (Particles/mL)	Notes/Comments
Arntz et al., 2015 ^16^	Cow (semiskimmed, UHT)	Differential ultracentrifugation +0.2 μm syringe Filtration	DLS/NTA	2.5 × 10^7^	Very low recovery
Santos-Coquillat et al., 2021 ^17^	Goat (UHT)	Differential ultracentrifugation + SEC	DLS/NTA	0.5–1.1 × 10^9^ partículas/mL	Chromatographic enrichment
Vaswani et al., 2021 ^18^	Cow (unpasteurized)	Differential ultracentrifugation + SEC	NTA	1.4 × 10^11^	High particle recovery with enrichment
Present study (2025)	Cow (UHT)/goat (UHT)	Differential ultracentrifugation	NTA	9.3 × 10^9^ (Cow)	Highest yield reported among comparable studies/aligned with enriched methods
				7.0 × 10^9^ (Goat)	

a Comparison of particle concentrations determined by NTA and DLS for exosomes isolated from different milk sources. Results emphasize the influence of the isolation method on nanoparticle recovery.

These results suggest that while simplified methods favor scalability and ease of processing, the incorporation of additional enrichment steps may enhance nanoparticle recovery by improving the selectivity and concentration of vesicular fractions. Therefore, the use of streamlined ultracentrifugation-based protocols offers a practical balance between yield and scalability, reinforcing milk as a cost-effective and abundant bioresource for exosome production in drug delivery applications.

Next, the morphological analysis performed by scanning electron microscopy (SEM) (Figure 1A and B) revealed vesicles with predominantly spherical structures for both samples. The same shape was also observed by other authors who analyzed bovine milk exosomes using a corresponding methodology: transmission electron microscopy (TEM).^19,20^ Similarly, when analyzing nanoparticle diameters by the NTA technique, no statistically significant variation was found between the two species (CM: 67.4 nm ± 2.4 nm; GM: 80.8 ± 3.3 nm) (Figure 1C). This result is consistent with Ahmed and colleagues’ previous publication. Despite finding larger diameters, the authors also did not observe significant differences in the size of exosomes isolated from cow, goat, and buffalo milk.^14^

**Figure 1:**
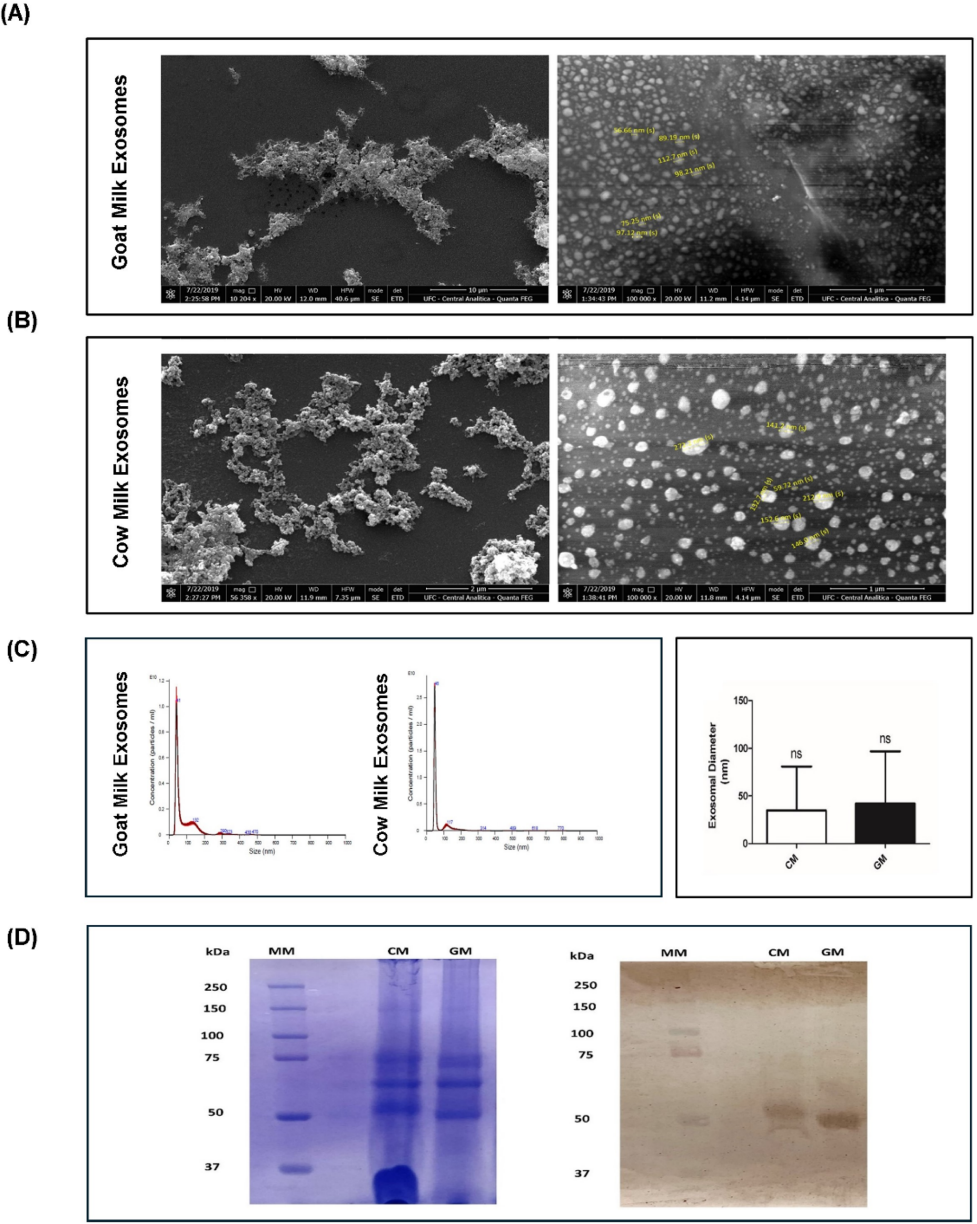
Morphological characterization of cow and goat milk exosomes using SEM; NTA and Western Blot. (A) Scanning Electron Microscopy (SEM): images illustrating morphology and size distribution of goat milk exosomes at 10,000× and 100,000× magnification; (B) cow milk exosomes at 10,000 and 100,000× magnifications. Representative nanoparticle diameters highlighted yellow. (C) Nanoparticle Tracking Analysis (NTA) with average size distribution of goat milk exosomes and cow milk exosomes, and comparison of mean diameters between species (mean ± SEM; Student’s *t*-test, *p* < 0.05). (D) SDS-PAGE and Western Blot detection of HSP70, confirming the presence of the exosomal marker. MM: molecular weight marker; CM: cow milk exosomes; GM: goat milk exosomes.

Thereafter, exosomes were further characterized regarding the presence of the established membrane marker, the cytoplasmic protein HSP70. SDS-PAGE analysis revealed multiple protein bands, including some around 50 kDa (Figure 1D), consistent with the expected molecular weight of heat shock protein 70 (approximately 53–70 kDa).^21^ Finally, the presence of the protein was confirmed by Western Blot immunodetection (Figure 1D).

The heat shock proteins are a family of molecules involved in the maintenance of cellular homeostasis under stressful conditions and can be mostly found in the cytosol or associated with microfilaments in cell surface projections.^21^ Exosomes differ from microvesicles and apoptotic bodies not only in terms of size but also in their formation process. Exosomes originate from an invagination process of the parental plasma membrane, which permits direct contact with the cytoplasm and its content. In contrast, the other extracellular vesicles (EVs) are formed through outward budding and fission from plasma membranes.^22^ So, this is the reason why exosomes are the only ones that contain heat shock proteins in their structures.

After the isolation step and the exosomes were obtained, tests were performed to load them with biomolecules, in this case, with an *M. oleifera* protein. In similar research, Haney et al.^23^ also assessed the efficacy of loading exosomes with a protein using passive or active techniques: incubation at room temperature with or without saponin, freeze–thaw cycles, sonication, and extrusion. Their findings led to the conclusion that the most effective techniques were the active ones (freeze–thaw cycles, sonication, extrusion, and incubation at room temperature in the presence of saponin).

*Mo*-CBP_3_ is a 2S albumin isolated from the *M. oleifera* tree seed and was chosen because it is a chemically and morphologically very well-characterized molecule.^24^ The encapsulation of hydrophilic particles, such as *Mo*-CBP_3_, presents a greater challenge due to their lower affinity for the hydrophobic exosomal membrane. Therefore, only active encapsulation methodologies were tested in the present study.

Three of the four methods used (electroporation [E], sonication [S], and freeze–thaw cycles [FC]) operate on essentially the same principle: disruption of exosome membrane homeostasis, either by exposure to electric current, sound waves, or sudden temperature changes. The incubation at room temperature [RT] was performed only in the presence of saponin, which also transiently alters the composition of the exosome membrane. Saponin not only creates temporary pores but also removes cholesterol, reducing bilayer stiffness and promoting greater interactions between the membrane and proteins.^11^ Notably, the incubation time (18 h—adapted from Haney et al.) was substantially longer than that reported in other studies (5–15 min).^25,26^

To assess the efficiency of *Mo*-CBP_3_ encapsulation, fluorescence spectrophotometry was initially performed. The results of the fluorescence intensity analysis demonstrated that both goat- and cow-derived exosomes effectively encapsulated *Mo*-CBP_3_–FITC. No differences were detected among the loading methodologies in the interspecies analysis. However, the intraspecies comparison revealed that freeze–thaw cycle goat milk exosomes demonstrated superior performance compared to the electroporated ones (Figure 2A). Subsequently, the loading efficiency (%LE) observation revealed no differences among the encapsulation methodologies tested. Both intra- and interspecies comparisons indicated comparable loading capacities for all tested approaches (Figure 2B).

**Figure 2:**
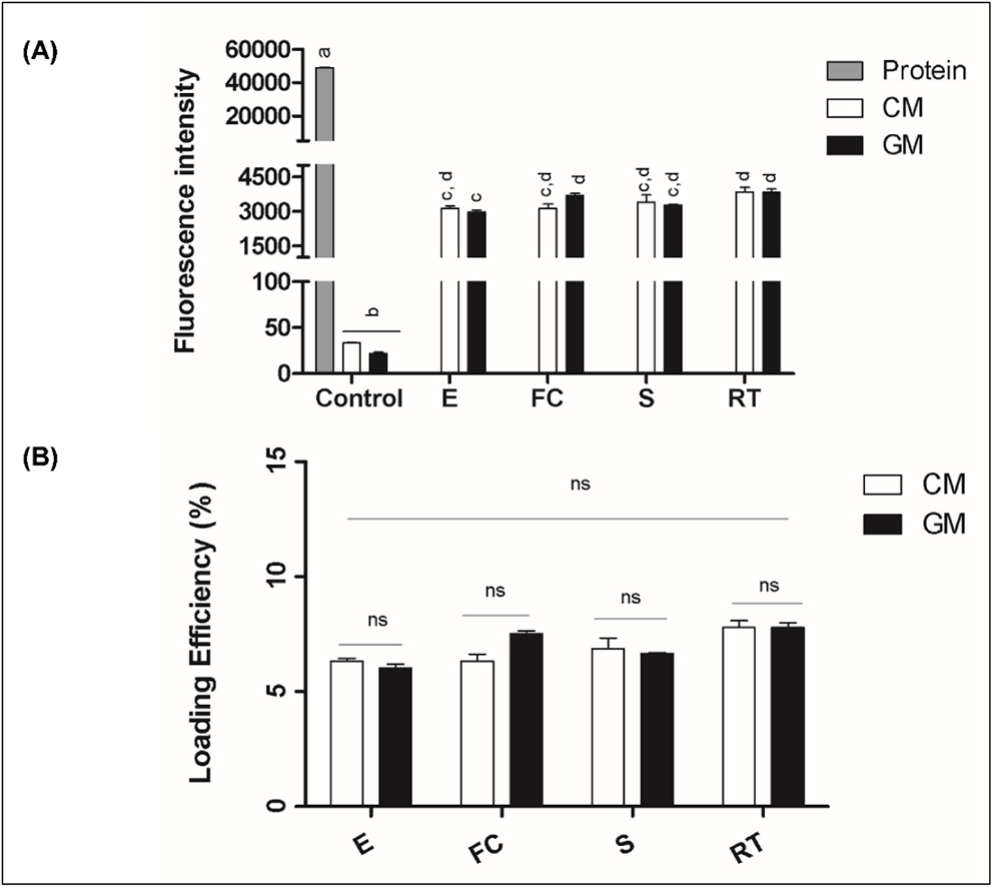
Fluorescence-based quantification and loading efficiency of the *Mo*-CBP_3_–FITC protein in milk-derived exosomes. (A) Fluorescence intensity of exosomes isolated from cow’s milk (CM, white bars) and goat’s milk (GM, black bars) loaded with *Mo*-CBP_3_–FITC protein by different methods: incubation at room temperature (RT), freeze–thaw cycles (FC), sonication (S), and electroporation (E). Gray bars represent the free (unencapsulated) *Mo*-CBP_3_–FITC protein, followed by unloaded (negative control) CM and GM exosomes. Different letters indicate statistically significant differences (*p* < 0.05). (B) Corresponding loading efficiency (%) of the *Mo*-CBP_3_–FITC protein calculated from fluorescence data after subtraction of background fluorescence from unloaded (negative control) samples. The interspecies analysis revealed no significant differences across loading methodologies (Tukey’s multiple comparison, *p* > 0.05).

Statistical analysis was performed using one-way ANOVA followed by Tukey’s multiple-comparison test (*p* < 0.05). Light and dark bars represent distinct samples (cow and goat milk), not biological replicates. NS: nonsignificant.

The average loading efficiencies obtained in this study (6–8%) were consistent with previously reported data for exosomal encapsulation of protein cargos. Earlier research described loading efficiencies ranging from approximately 5–30% for a similar protein incorporated into exosomes through room-temperature incubation for 1 h.^27^

Interestingly, Ahmed et al. , using the same exosomal sources—goat and cow milk—and fluorescence spectrophotometry to quantify encapsulation efficiency, reported loading efficiencies of 5–10%, closely matching those obtained in the present study. Notably, their findings demonstrated that these encapsulation levels were sufficient to promote effective intracellular delivery of the drug, indicating that relatively modest loading efficiencies can still achieve satisfactory therapeutic outcomes.^14^

Thereafter, electrophoresis and Western Blot were used to assess the protein encapsulation efficiency under different methodologies. Upon electrophoresis in denaturing conditions (SDS-PAGE), *Mo*-CBP_3_ separates into two protein bands with apparent masses of 28 and 17 kDa.^24^ Such a pattern was observed in the samples from the loaded exosomes as well as in the positive controls (in which the naive protein was subjected to electrophoresis alone), whereas in the negative controls (unloaded exosomes from CM and GM), this pattern was not found (Figure 3A,C). The success of exosome loading was then confirmed by performing WB (Figure 3B,D).

**Figure 3:**
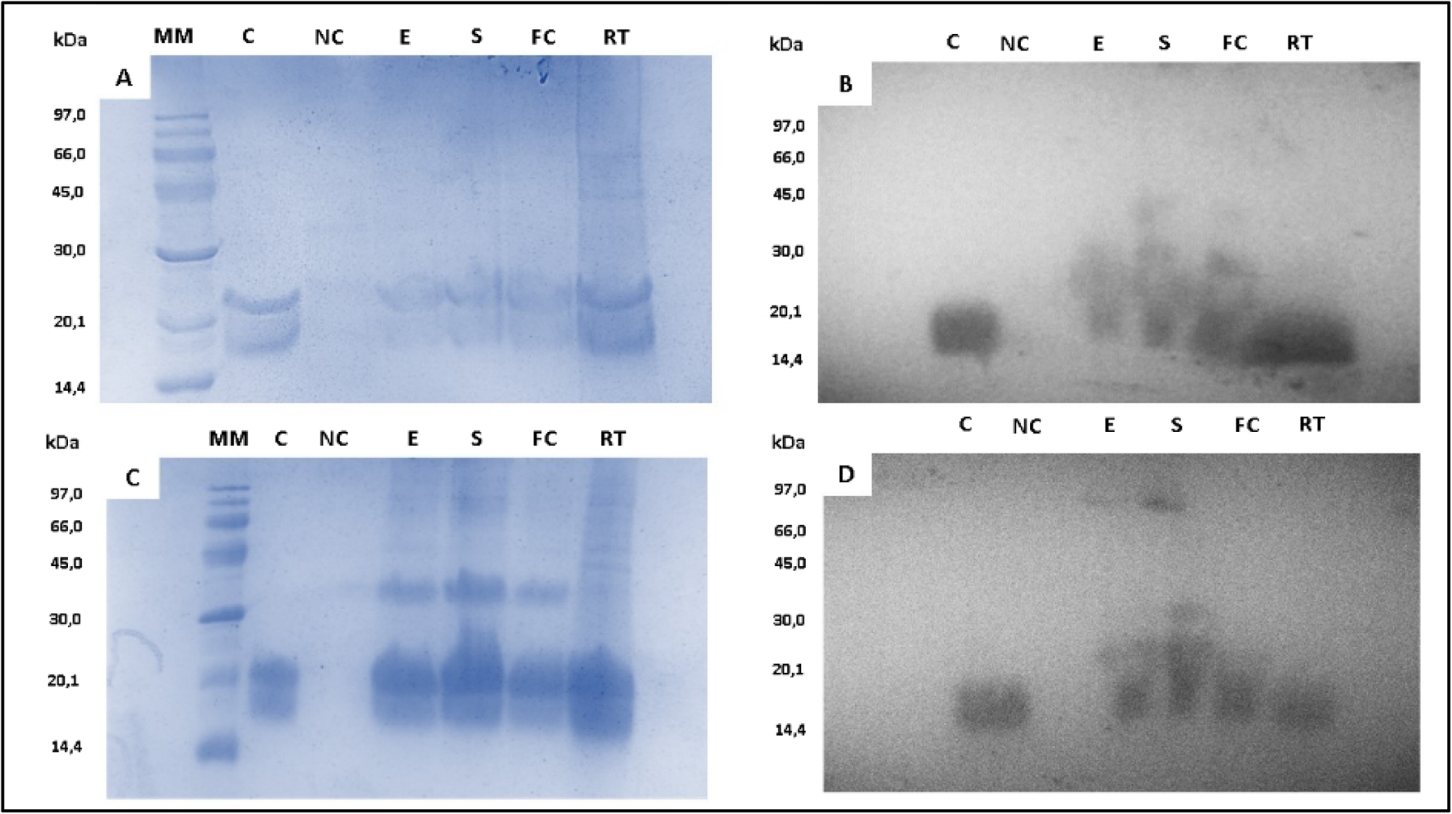
Analysis of *Mo*-CBP_3_ protein encapsulation by electrophoresis and Western Blot. *Mo*-CBP_3_ loading on exosomes was confirmed by polyacrylamide gel electrophoresis (15%) for goat (A) and cow (C) milk samples. Western Blot of the same samples demonstrated the efficacy of *Mo*-CBP_3_ loading protein into goat (B) and cow (D) milk exosomes. MM: molecular weight marker; C: positive control (*Mo*-CBP_3_, 10 μg); NC: negative control (empty exosome proteins); exosomal proteins (10 μg) after *Mo*-CBP_3_ exosome charging by electroporation method (E); sonication (S), freeze–thaw cycles (FC), and incubation at room temperature (RT).

As previously mentioned, the hydrophilic nature of the *Mo*-CBP_3_ molecule enhances its responsiveness to active loading methods, as it inherently lacks an affinity for the lipid bilayer of the exosomal membrane. Furthermore, all methods employed—electroporation, sonication, freeze–thaw cycles, and incubation with saponin at room temperature—operate via essentially the same mechanism. This likely explains the high loading capacity observed across all four methods in both CM and GM samples.

The next step was the cell incubation assay (Figure 4A,B). The results revealed that the endocytosis of loaded exosomes varied in efficiency across the different loading methods in both species. For cow milk exosomes, electroporation was the only method that appeared ineffective in cargo delivery (Figure 4B). According to Johnsen et al.,^28^ electroporation is a great option for exosome loading, although it can be related with many adverse effects on particle integrity and therapeutic cargo. Another author found that the electroporation process induces nucleic acid (siRNA) aggregation, compromising its loading into extracellular vesicles.^29^ Electroporation also appears to promote exosome particle aggregation, which further impairs drug loading and their consequent use as biopharmaceuticals.^30^

**Figure 4:**
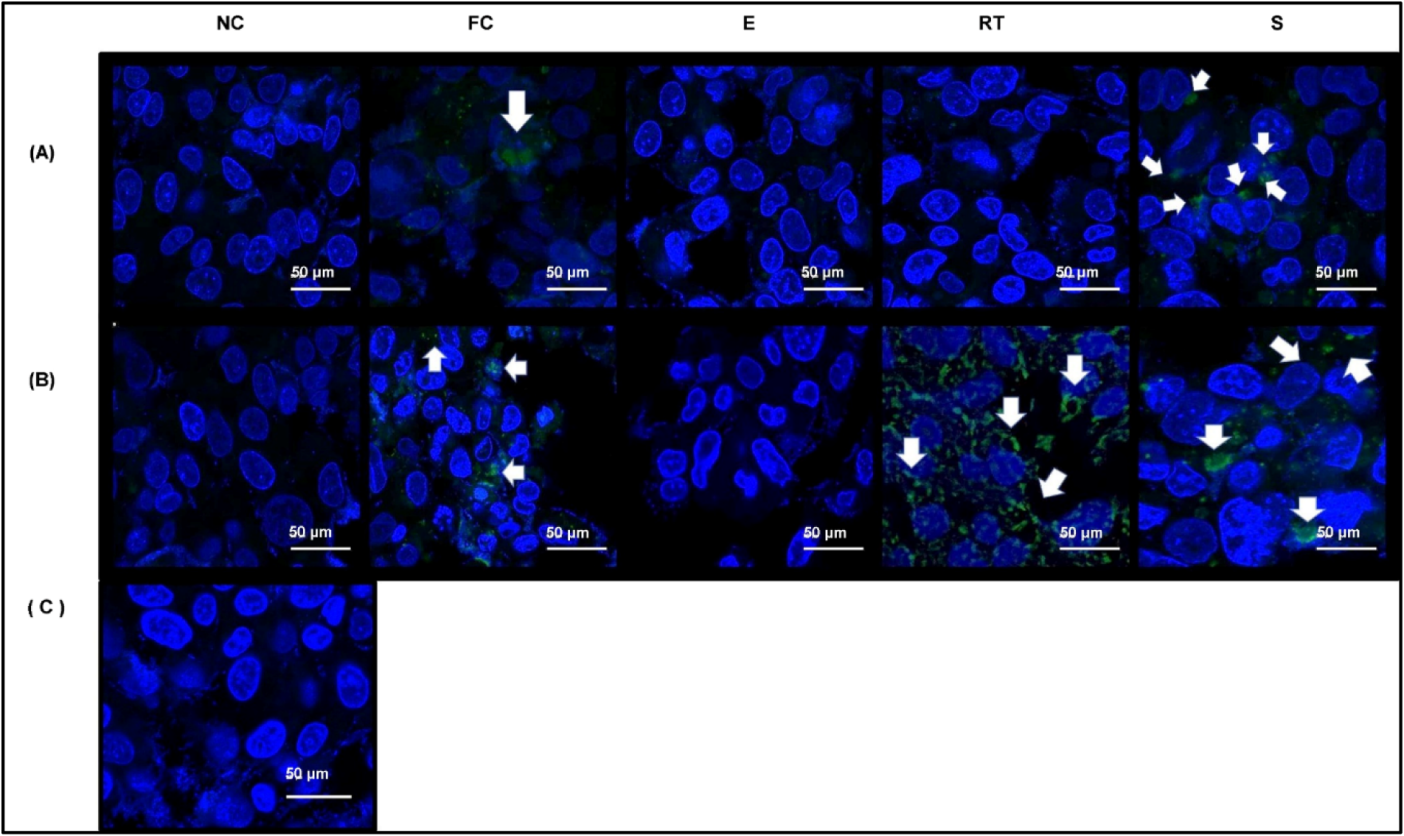
Confocal microscopy analysis of the exosome uptake by HepG2 cells. (A,B) Internalization of goat (A) and cow (B) milk exosomes loaded with *Mo*-CBP_3_ protein conjugated to FITC (green, indicated by white arrows) after 24 h of incubation. Nuclei were counterstained with DAPI (blue). Scale bars: 50 μm. Negative control (C) consisted of HepG2 cells cultured for 24 h in DMEM with unloaded exosomes.

In this study, SEM analysis revealed that electroporated exosomes exhibited morphological changes, including irregular shapes, membrane disruption, and small cluster formation, likely induced by electric field exposure (Figure 5A,B). In contrast, native exosomes maintained uniform and spherical morphology. (Figure 1A,B).

**Figure 5:**
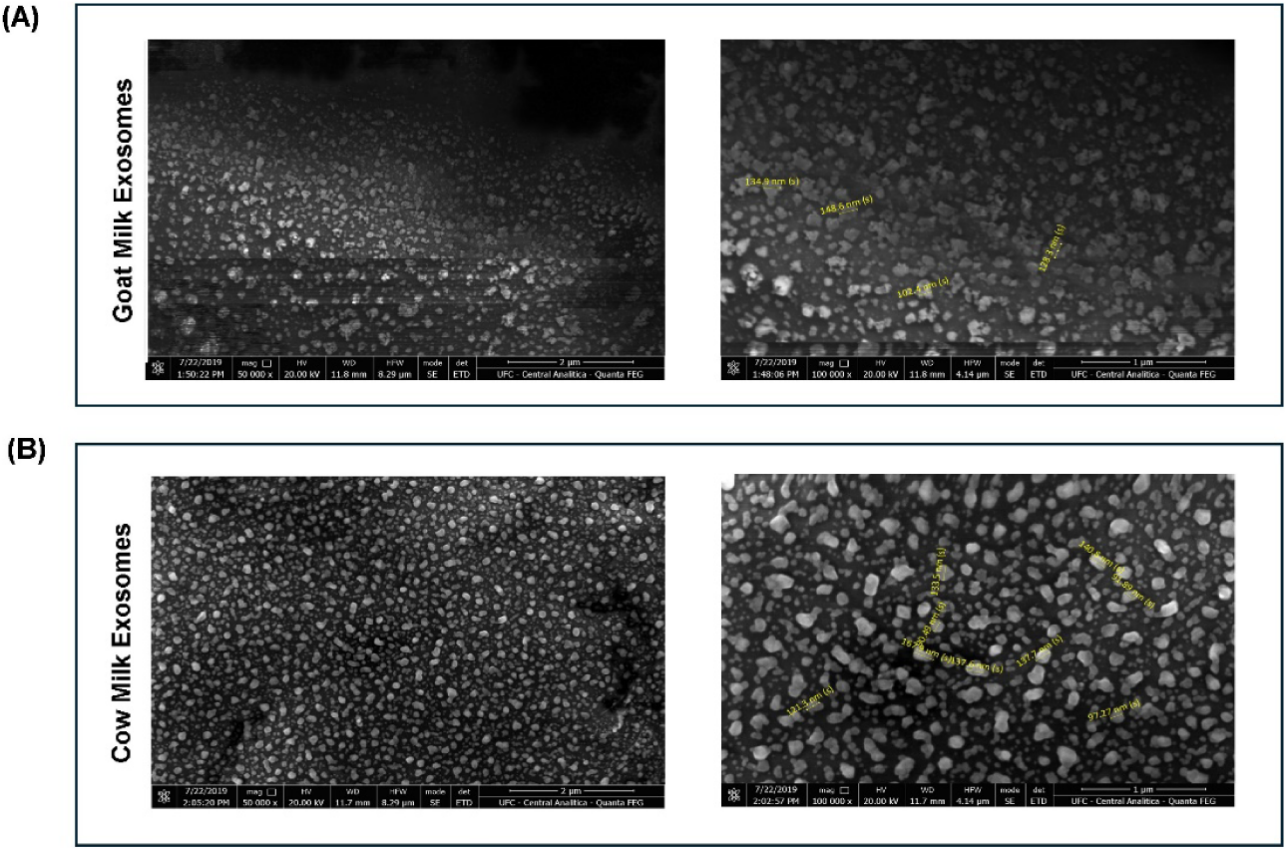
Scanning Electron Microscopy of goat and cow milk exosomes after electroporation. (A) Representative images of goat milk exosomes at 50,000× and 100,000× magnifications. (B) Morphology and size of cow milk exosomes at 50,000× and 100,000× after electroporation. Yellow markers indicate representative nanoparticle diameters.

In addition, as reported by Kooijmans et al., the aggregation effect induced by electroporation can lead to errors in spectrophotometric measurements, since the device cannot distinguish between fluorescence emitted by loaded exosomes and that from insoluble aggregates of unencapsulated molecules. This phenomenon may give a misleading impression of encapsulation efficiency, explaining the discrepancies observed between the loading efficiency data and the unsuccessful cargo delivery to HepG2 cells in both CM and GM samples. Consequently, this suggests that spectrophotometric analysis may overestimate the loading capacity of electroporated exosomes and, for this reason, should not be considered a reliable approach for assessing nanoparticles previously subjected to electroporation procedures.^29^

Another discrepancy evidenced between the loading efficiency analysis and the results of the cell incubation assay concerns saponin-assisted incubation (RT). In the same way, although this method efficiently loaded both samples, goat milk exosomes failed to deliver their cargo to the HepG2 cells. This outcome is likely to be multifactorial. A prolonged incubation exposure (18 h) and the naturally higher membrane fatty acid content of goat milk exosomes^14^ may have irreversibly damaged the vesicle integrity, leading to continuous leakage of the encapsulated protein until complete depletion of the cargo before the *in vitro* tests. Alternatively, such membrane alterations could have impaired exosome–cell interactions, ultimately compromising the internalization and molecule delivery.

Conversely, while our results revealed slight differences in delivery capacities depending on milk source and loading method, Ahmed et al. reported that goat milk exosomes were more efficient than bovine and buffalo milk exosomes in loading the chemotherapeutic doxorubicin, likely due to their higher membrane polyunsaturated fatty acid content, which confers greater flexibility under chemical and physical stimuli.^14^

Functionally, goat milk exosomes demonstrated superior performance both as carriers of doxorubicin (DOXO) and in their native (unloaded) form. They not only provided greater drug stability and improved antitumor effects compared to free DOXO but also reduced tumor cell viability on their own. These effects have been associated with the presence of specific fatty acids, such as capric, caprylic, and caproic acids, which have been linked to tumor cell death in previous studies.^14^

Like all other pharmaceutical processes, drug loading methods must be scalable and have their validation established. The morphological alterations and aggregation induced by electroporation can significantly hinder the scalability of exosome-based drug delivery systems,^31^ since such structural instabilities require additional purification steps to isolate functional exosomes, thereby increasing production costs and complexity.^29^ Furthermore, these morphological changes can affect the pharmacokinetics and biodistribution of exosomes *in vivo.*

Aggregated exosomes may exhibit altered interactions with biological membranes, potentially leading to reduced cellular uptake and premature clearance from circulation, which diminishes their therapeutic efficacy.^31^ Therefore, while electroporation remains a widely used method for loading biomolecules into exosomes, its associated challenges highlight the need for optimization to enhance the scalability and effectiveness of this exosome-based therapy *in vivo.*

According to Kalani et al., the freeze–thaw cycling (FC) method is a great alternative for loading and evaluating the *in vivo* efficacy of exosomes. The authors found that many membrane proteins were still functional even after performing this method, indicating maintenance of structural integrity and function. In addition, these exosomes were able to produce beneficial effects in the reversal of brain injury caused by ischemia followed by reperfusion.^25^ Consistent with our results, where FC goat milk exosomes exhibited superior fluorescence among the tested methods and both FC samples (GM and CM) were effective in delivering the cargo to HepG2 cells, this approach emerges as a promising candidate for future studies, especially *in vivo*.

Finally, sonicated exosomes from both goat (GM) and cow (CM) milks exhibited an enhanced capacity for cargo delivery to target cells. Similar observations were reported by Li et al. when analyzing the encapsulation efficiency of hydrophilic molecules—comparable in nature to *Mo*-CBP_3_—in cow milk exosomes. The authors demonstrated that sonication and saponin-assisted incubation were the most effective loading methodologies. However, when progressing to *in vitro* assays, the saponin-supplemented incubation showed high cytotoxicity, limiting its applicability.^32^

Similarly, Ahmed et al. used the same exosomal sources—cow and goat milk—to evaluate the encapsulation capacity of doxorubicin, a small hydrophilic chemotherapeutic. Goat milk exosomes showed superior loading efficiency, while room-temperature or saponin-assisted incubation achieved the highest loading rates. However, due to the well-known cytotoxicity of saponin, the authors proceeded only with the room-temperature method for subsequent *in vitro* experiments.^14^

In addition, Haney et al. demonstrated that sonication was the most effective method for encapsulating and delivering the hydrophilic macromolecule catalase into neuronal cells using macrophage-derived exosomes. The authors attributed this superior performance to a reorganization of exosomal membrane proteins and bilayer lipids induced by the sonication process, which increases the vesicle surface area and promotes stronger interactions with the target cells. However, this same membrane disruption may compromise the natural immunological privileges of exosomes, potentially enhancing their recognition and clearance by the mononuclear phagocyte system.^23^

Finally, milk-derived exosomes seem to display low immunogenicity and no detectable toxicity, even after oral administration.^21,33,34^ Nevertheless, they can enhance immune activity under preexisting inflammatory conditions, as shown by Komine-Aizawa et al., who observed increased IFN-γ production by NK cells upon costimulation with IL-2 and IL-12, compared with the control.^35^ In addition, it is speculated that milk exosomal macromolecules, such as miRNAs and mRNAs, can play a critical role in the development and modulation of the immune system.^15^ Therefore, factors such as lactation stage and milk processing can cause variability in bioactive cargo, potentially affecting the functional consistency of exosomes *in vivo*.^36^

## 3 Conclusions

This study provides the first comprehensive evaluation of a hydrophilic biomacromolecule loaded into milk-derived exosomes through four distinct encapsulation strategies (previous studies focused on small drugs or nonmilk exosomal sources). To the best of our knowledge, this is the first work to systematically cross-examine four encapsulation approaches within this specific system, thereby providing novel experimental evidence in the field of exosome-mediated drug delivery.

Among the tested methods, sonication and freeze–thaw cycles yielded the most efficient encapsulation and delivery to HepG2 cells for both GM and CM milk exosomes, while also offering practical advantages such as simplicity, speed, and scalability—features that make them promising for large-scale biopharmaceutical development. Importantly, cow milk exosomes proved more effective in cargo delivery to the tested cell line, as three of the four loading methods achieved successful delivery; however, further investigations involving additional cell lines and *in vivo* assays are warranted to confirm and extend these findings.

Finally, despite the favorable performance of sonication and freeze–thaw cycles, potential structural alterations caused by sonication highlight the need for future *in vivo* studies to ensure their translational potential as efficient nanocarriers for macromolecular therapeutics.

## 4 Materials and Methods

### 4.1 Isolation of Exosomes from Cow and Goat Milk

UHT (ultrahigh temperature) milk from cow and goat was purchased from local markets and used as the source for exosome isolation. Three independent samples of each species were distributed and centrifuged in 38.5 mL tubes using a Beckman Coulter Optima XE-90 ultracentrifuge. Milk was first centrifuged for 35 min, at 13,200*g*, 4 °C, to precipitate debris and somatic cells. The supernatant was then centrifuged for 60 min, at 30,000*g*, 4 °C, to remove precipitated proteins, fat globules, and vesicles larger than exosomes. To remove casein aggregates, the suspension was ultracentrifuged at 80,000*g*, 4 °C, for 60 min. Finally, to collect the exosomes, the supernatant was ultracentrifuged at 120,000*g*, 4 °C, for 90 min. After all these steps, the exosome-containing precipitate was resuspended in a minimal volume of phosphate-buffered saline (PBS) solution and then stored at −80 °C. This methodology was adapted from the protocol described by Kusuma et al.^20^ No major differences were detected among the various milk batches used in this research.

### 4.2 Protein Determination

The membrane protein concentration in the exosomes was quantified using the Bradford protocol adapted for microplates.^37^ The exosome samples were diluted 50-fold and compared in duplicate against a serially diluted bovine serum albumin standard, following the manufacturer’s recommendations (Bio-Rad). The absorbance of the samples was analyzed using a Synergy 2 microplate reader (Biotek, USA) at 595 nm, and the values were extrapolated from the standard curve.

### 4.3 Morphological Characterization of Exosomes

#### 4.3.1 Scanning Electron Microscopy (SEM)

Exosomes were characterized for their size, distribution, and morphology by scanning electron microscopy (SEM). For sample preparation, exosomes were fixed in Karnovsky’s solution [1% glutaraldehyde (v/v) + 4% paraformaldehyde (v/v) prepared in 0.15 M sodium phosphate buffer, pH 7.2] and transferred to coverslips coated with 1% gelatin. They were then treated with 0.1% (v/v) osmium tetroxide and later washed in PBS buffer. After washing, samples were dehydrated through a series of ethanolic solutions (10%, 30%, 50%, 70%, 90%, and 100%), followed by immersion in a 50/50 (v/v) ethanol/hexamethyldisilazane (HMDS) solution and 100% HMDS (10 min incubation under each condition). A thin layer of gold was applied using a metallizer to enhance visibility. Subsequently, images were captured using a Scanning Electron Microscope (SEM) (Quanta 450 FEG—FEI) at 20 kV with magnifications of 10,000× and 100,000×, and 50,000x, and 100,000x.

#### 4.3.2 Nanoparticle Tracking Analysis (NTA)

The size distribution and concentration of purified exosomes from goat and bovine milk were determined using Nanoparticle Tracking Analysis (NTA) on a Nanosight NS500 instrument (software version NTA 3.4 Build 3.4.4). For each measurement, 10 μL of the exosomal suspension, diluted in ultrapure water, was analyzed in triplicate.

### 4.4 Exosomal Marker Detection by Western Blot

Bovine and caprine milk were analyzed for the presence of the cytoplasmic heat shock protein HSP70 by Western Blot (WB). Samples (20 μL; approximately 100 μg of protein/well) were loaded onto a 15% polyacrylamide gel (SDS-PAGE) and subjected to electrophoresis (400 mA, 160 V for 2 h) under denaturing conditions [2% (v/v) β-mercaptoethanol: sample buffer]. The proteins were then transferred to a nitrocellulose membrane in a semidry electrotransfer apparatus using the transfer buffer (39 mM glycine, 0.03% SDS (w/v); 20% (v/v) methanol; 48 mM Tris, pH 8.0) at a constant current of 56 mA, 30 V for 3 h. The nonspecific sites of the membrane were blocked with 5% (w/v) BSA and 0.05% Tween 20 (v/v) in PBS buffer for 1 h at room temperature. The mouse monoclonal anti-Hsp70 primary antibody (diluted 1:1000 in PBS-T) was incubated overnight at 4 °C in a shaker device, and after washing, the HRP-conjugated secondary antibody (diluted 1:6000 in PBS-T) was added for 1 h at room temperature. Finally, the immunoreactive bands were visualized using a DAB (3,3′-diaminobenzidine tetrahydrochloride) solution.

### 4.5 Conjugation of *Moringa oleifera* 2S Albumin (*Mo*-CBP_3_) with Fluorescein Isothiocyanate (FITC)

The 2S albumin *Mo*-CBP_3_ (*M. oleifera*-Chitin-binding protein) was kindly provided by the Plant Toxin Laboratory of the Department of Biochemistry and Molecular Biology of the Federal University of Ceará. From this, a standard solution of 4 mg/mL was prepared in 0.1 M sodium carbonate buffer, pH 9.0. Subsequently, FITC was dissolved in anhydrous DMSO at a concentration of 1 mg/mL. To each milliliter of the protein solution, 100 μL of the fluorophore solution was added in 5 μL aliquots. The conjugate was then incubated overnight at 4 °C in the dark. The next day, ammonium chloride (final concentration 50 mM) was added to the solution and incubated for a further 2 h, at 4 °C to stop the conjugation reaction. Finally, the unconjugated fluorophore was separated on a gel filtration column. The protein-fluorophore conjugation protocol was performed according to the manufacturer’s instructions (Sigma-Aldrich). The final concentration of the conjugated protein was determined by measuring the fluorescence intensity of the sample by fluorescence spectrophotometry (excitation wavelength 495 nm and emission wavelength 525 nm) using the Synergy 2 microplate reader (Biotek, USA).

### 4.6 *Mo*-CBP_3_ Loading in Cow and Goat’s Milk Purified Exosomes

The active loading methodologies consist of techniques that cause transient disruption of the exosomal membrane. In this study, four different active loading techniques were tested: (1) electroporation, (2) sonication, (3) freeze–thaw cycles, and (4) incubation at room temperature in the presence of saponin (0.2%). A 1:1 ratio of exosomal protein and *Mo*-CBP_3_ was observed in all treatments (500 μg each).

For method 1, solutions were prepared in a final volume of 200 μL according to the concentrations and ratios described above. They were then transferred to 4 mm cuvettes, previously cooled to 4 °C, and subjected to electroporation according to the following parameters: (450 V, 2 pulses of 15 ms). For this methodology, the Multiporator electroporator (Eppendorf, Germany) was used, and the procedure was adapted from Tian et al.^38^ For method 2, the mixture of exosomes and *Mo*-CBP_3_ was first sonicated (6 cycles of 2 s pulses with10 s pauses) and then cooled on ice for 2 min. The above procedure of sonication was repeated twice for each sample. Sonication was performed using a Q55 Sonicator (QSonica, LLC, Newtown, CT, USA) equipped with a titanium probe. The device operates at a fixed nominal power of 55 W and a frequency of 20 kHz, parameters that cannot be adjusted by the operator. Consequently, all samples were processed under identical power and frequency settings. For method 3, the exosome and *Mo*-CBP_3_ solution was first incubated at room temperature for 30 min, then rapidly frozen in liquid nitrogen, followed by thawing at room temperature. This freeze–thaw cycle was repeated three times for each sample. Finally, for method 4, saponin was added to the mixture of exosomes and *Mo*-CBP_3_ for a final concentration of 0.2%. The mixture was vortexed for 20 min and then incubated at room temperature for 18 h.

It ís worth noting that, after electroporation and sonication, samples were incubated at 37 °C for 30 min to ensure complete recovery of the exosomal membrane from the transient pore formation induced by exposure to electrosonic waves. These three last methodologies were adapted from the protocols described by Haney et al.^23^

### 4.7 *Mo*-CBP_3_ Loading Detection

The efficacy of the loading methods was confirmed by fluorescence spectrophotometry and Western Blot. For technique 1, 100 μL of each sample (exosomes loaded with the FITC-*Mo*-CBP_3_ using the four different methods) was loaded onto 96-well plates (experiments were performed in duplicate), followed by analysis (emission at 525 nm and excitation at 495 nm). To enable technique 2, the protein previously loaded into exosomes (and not conjugated to FITC) was extracted by using freeze–thaw cycles and then precipitated with 12.5% trichloroacetic acid (TCA). Then, electrophoresis (400 mA and 180 V for approximately 1 h) was performed on a polyacrylamide gel (SDS-PAGE) with 8 μL of each sample/well (including positive and negative controls). After separation by SDS-PAGE, proteins were transferred to a polyvinyl difluoride (PVDF) membrane in a semidry electrotransfer device using the transfer buffer 3-(cyclohexylamino)-1-propanesulfonic acid (CAPS) at a constant current of 120 mA for 30 min. Nonspecific sites on the membranes were blocked overnight with a PBS solution containing 5% skimmed milk (w/v). Incubation with the primary (rabbit polyclonal anti-*Mo*-CBP_3_) and secondary (rabbit anti-IgG) antibodies was performed at a dilution of 1:5000 for 1 h at room temperature. Finally, the protein bands from the print were visualized by immersing the membranes in a DAB solution.

The loading efficiency (LE%) determination of FITC-labeled MoCBP_3_ (MoCBP_3_–FITC) was evaluated by ratiometric fluorescence analysis. After the loading procedures, exosome samples were subjected to an additional ultracentrifugation step (120,000*g*, 4 °C, 90 min) to remove unbound MoCBP_3_–FITC. Fluorescence intensities were measured in duplicate using a microplate reader (excitation/emission: 495/525 nm). LE% was calculated by using the following equation:

$$\%\mathrm{LE}=\left(\frac{F_{\mathrm{Loaded}\;\mathrm{exosomes}}-F_{\mathrm{Control}}}{F_{\mathrm{Protein‐MoCBP3}}}\right)\times 100$$

Where *F* represents the fluorescence intensity for each respective sample.

*F*_loaded exosomes_: fluorescence intensity from different tested methodologies

*F*_control_: mean fluorescence intensity from unloaded exosomes (negative control)

*F*_protein–*Mo*CBP3:_ mean fluorescence intensity from free *Mo*CBP_3_–FITC

### 4.8 Uptake of *Mo*-CBP_3_-Encapsulated Exosomes

HepG2 cells (human hepatocarcinoma) were generously provided by Dr. Rita Negrão from the Biochemistry Unit of the Biomedicine Department at the Faculty of Medicine, University of Porto, and were maintained at the Laboratory of Biotechnology and Molecular Biology (LBBM) at the State University of Ceará (UECE). The culture medium used (DMEM without phenol red) was supplemented with 10% fetal bovine serum, sodium bicarbonate, glutamine, antibiotics, and antifungals (Vitrocell Embriolife, Nova Campinas, Brazil). Prior to incubation, all samples of loaded exosomes were washed by ultracentrifugation at 120,000*g* for 1.5 h. This step was performed to remove PBS and avoid its interference in subsequent analysis by confocal microscopy. Samples were then resuspended in the same culture medium described above to perform the incubation assay. HepG2 cells were distributed on circular coverslips in 24-well plates (1 × 10^5^ cells per well) and incubated in a CO_2_ incubator (Sanyo MCO-19AIC­(UV), Japan) (37 °C and 5% CO_2_) until reaching 70% confluence. Then, each sample of exosomes (corresponding to the different loading methodologies) was added to the wells (observing the concentration of 0.1 μg/μL) and incubated for 24 h. The tests were also performed with positive and negative controls (tests were performed in duplicate).

### 4.9 Analysis by Confocal Microscopy

After the cell incubation assay, the culture medium was removed, and cells were washed three times in 20 mM sodium phosphate buffer, pH 7.4, and fixed with paraformaldehyde solution (4%) for 30 min. After that, samples were rewashed in sodium phosphate buffer and incubated with 4',6'-diaminido-diamidino-2-phenylindole (DAPI) dye for 15 min for nuclei detection. Finally, samples were washed three times in a 0.9% NaCl solution and then analyzed by confocal microscopy (LM710-Confocal – Zeiss, Germany) with a laser at 488 nm. Images were processed, and fluorescence was quantified using Alphaview software (ProteinSimple, USA).

### 4.10 Statistical Evaluation

All data were analyzed using the GraphPad Prism 6.0 software. Statistical significance was determined by *p* < 0.05, and three tests were performed for the *p*-values: the Student́s *t*-test (Figure 1C) and one-way ANOVA followed by Tukey test (Figure 2). All results were presented as the mean ± standard deviation.

## 5 Highlights

Exosomes were successfully isolated from both goat milk (GM) and cow milk (CM). A chitin-binding protein was effectively charged into exosomes from GM and CM. All methods of protein charging were effective. Exosomes loaded with Mo-CBP_3_ were endocytosed by HepG2 cells (human hepatocarcinoma).

## References

[1] Wang X., Thomsen P. (2021). Mesenchymal stem cell–derived small extracellular
vesicles and bone regeneration. Basic Clin.
Pharmacol. Toxicol..

[2] Cho Y. E., Song B. J., Akbar M., Baek M. C. (2018). Extracellular vesicles
as potential biomarkers for alcohol- and drug-induced liver injury
and their therapeutic applications. Pharmacol
Ther..

[3] Yan M., Zhang X., Pu Q., Huang T., Xie Q., Wang Y., Li J., Wang Y., Gu H., Huang T., Li Z., Gu J. (2016). Immunoglobulin G Expression
in Human Sperm and Possible Functional Significance. Sci. Rep..

[4] Jeske R., Bejoy J., Marzano M., Li Y. (2020). Human pluripotent stem
cell-derived extracellular vesicles: Characteristics and applications. Tissue Eng. Part B Rev..

[5] Gutierrez-Millan C., Calvo Díaz C., Lanao J. M., Colino C. I. (2021). Advances
in Exosomes-Based
Drug Delivery Systems. Macromol. Biosci..

[6] Adriano B., Cotto N. M., Chauhan N., Jaggi M., Chauhan S. C., Yallapu M. M. (2021). Milk exosomes: Nature’s
abundant nanoplatform
for theranostic applications. Bioact Mater..

[7] Jafari D., Shajari S., Jafari R., Mardi N., Gomari H., Ganji F., Forouzandeh
Moghadam M., Samadikuchaksaraei A. (2020). Designer Exosomes:
A New Platform for Biotechnology Therapeutics. BioDrugs.

[8] Yáñez-Mó M., Siljander P. R. M., Andreu Z., Zavec A. B., Borràs F. E., Buzas E. I., Buzas K., Casal E., Cappello F., Carvalho J., Colás E., Cordeiro-Da Silva A., Fais S., Falcon-Perez J. M., Ghobrial I. M., Giebel B., Gimona M., Graner M., Gursel I., Gursel M., Heegaard N. H. H., Hendrix A., Kierulf P., Kokubun K., Kosanovic M., Kralj-Iglic V., Krämer-Albers E. M., Laitinen S., Lässer C., Lener T., Ligeti E., Line A., Lipps G., Llorente A., Lötvall J., Manček-Keber M., Marcilla A., Mittelbrunn M., Nazarenko I., Nolte-’t Hoen E. N. M., Nyman T. A., O’Driscoll L., Olivan M., Oliveira C., Pállinger É., Del Portillo H. A., Reventós J., Rigau M., Rohde E., Sammar M., Sánchez-Madrid F., Santarém N., Schallmoser K., Ostenfeld M. S., Stoorvogel W., Stukelj R., Van Der Grein S. G., Helena Vasconcelos M., Wauben M. H. M., De Wever O. (2015). Biological properties
of extracellular vesicles and their physiological functions. J. Extracell Vesicles.

[9] Larabi A., Barnich N., Nguyen H. T. T. (2020). Emerging
Role of Exosomes in Diagnosis
and Treatment of Infectious and Inflammatory Bowel Diseases. Cells.

[10] Kim H., Kim E. H., Kwak G., Chi S. G., Kim S. H., Yang Y. (2021). Exosomes: Cell-derived
nanoplatforms for the delivery of cancer therapeutics. Int. J. Mol. Sci..

[11] Fuhrmann G., Serio A., Mazo M., Nair R., Stevens M. M. (2015). Active
loading into extracellular vesicles significantly improves the cellular
uptake and photodynamic effect of porphyrins. J. Controlled Release.

[12] Zhuang X., Xiang X., Grizzle W., Sun D., Zhang S., Axtell R. C., Ju S., Mu J., Zhang L., Steinman L., Miller D., Zhang H. G. (2011). Treatment
of brain
inflammatory diseases by delivering exosome encapsulated anti-inflammatory
drugs from the nasal region to the brain. Mol.
Ther..

[13] Yang T., Martin P., Fogarty B., Brown A., Schurman K., Phipps R., Yin V. P., Lockman P., Bai S. (2015). Exosome delivered
anticancer drugs across the blood-brain barrier for brain cancer therapy
in Danio Rerio, Pharm Res. Pharm. Res..

[14] Ahmed F., Tamma M., Pathigadapa U., Reddanna P., Yenuganti V. R. (2022). Drug Loading
and Functional Efficacy of Cow, Buffalo, and Goat Milk-Derived Exosomes:
A Comparative Study. Mol. Pharm..

[15] Munagala R., Aqil F., Jeyabalan J., Gupta R. C. (2016). Bovine milk-derived
exosomes for drug delivery, Cancer Lett. Cancer
Lett..

[16] Arntz O. J., Pieters B. C. H., Oliveira M. C., Broeren M. G. A., Bennink M. B., De Vries M., Van Lent P. L. E. M., Koenders M. I., Van den
Berg W. B., Van der Kraan P. M., Van de Loo F. A. J. (2015). Oral
administration of bovine milk derived extracellular vesicles attenuates
arthritis in two mouse models. Mol. Nutr. Food
Res..

[17] Santos-Coquillat A., González M. I., Clemente-Moragón A., González-Arjona M., Albaladejo-García V., Peinado H., Muñoz J., Ximénez Embún P., Ibañez B., Oliver E., Desco M., Salinas B. (2022). Goat Milk Exosomes
As Natural Nanoparticles for Detecting Inflammatory Processes By Optical
Imaging. Small.

[18] Vaswani K., Mitchell M. D., Holland O. J., Qin Koh Y., Hill R. J., Harb T., Davies P. S. W., Peiris H. (2019). A Method for the Isolation
of Exosomes from Human and Bovine Milk. J. Nutr.
Metab..

[19] Gao, D. ; Jiang, L. Review Article Exosomes In Cancer Therapy: A Novel experimental Strategy, 2018. www.ajcr.us/. PMC6291654 30555736

[20] Kusuma R. J., Manca S., Friemel T., Sukreet S., Nguyen C., Zempleni J. (2016). Human vascular endothelial cells transport foreign
exosomes from cow’s milk by endocytosis. Am. J. Physiol. Cell Physiol..

[21] Li B., Hock A., Wu R. Y., Minich A., Botts S. R., Lee C., Antounians L., Miyake H., Koike Y., Chen Y., Zani A., Sherman P. M., Pierro A. (2019). Bovine milk-derived
exosomes enhance goblet cell activity and prevent the development
of experimental necrotizing enterocolitis. PLoS
One.

[22] Ha D., Yang N., Nadithe V. (2016). Exosomes as
therapeutic drug carriers
and delivery vehicles across biological membranes: current perspectives
and future challenges. Acta Pharm. Sin B.

[23] Haney M. J., Klyachko N. L., Zhao Y., Gupta R., Plotnikova E. G., He Z., Patel T., Piroyan A., Sokolsky M., Kabanov A. V., Batrakova E. V. (2015). Exosomes
as drug delivery vehicles for Parkinson’s
disease therapy. J. Controlled Release.

[24] Freire J. E. C., Vasconcelos I. M., Moreno F. B. M. B., Batista A. B., Lobo M. D. P., Pereira M. L., Lima J. P. M. S., Almeida R. V. M., Sousa A. J. S., Monteiro-Moreira A. C. O., Oliveira J. T. A., Grangeiro T. B. (2015). Mo-CBP3,
an antifungal chitin-binding protein from Moringa oleifera seeds,
is a member of the 2S albumin family. PLoS One.

[25] Kalani A., Chaturvedi P., Kamat P. K., Maldonado C., Bauer P., Joshua I. G., Tyagi S. C., Tyagi N. (2016). Curcumin-loaded
embryonic stem cell exosomes restored neurovascular unit following
ischemia-reperfusion injury. Int. J. Biochem.
Cell Biol..

[26] Sun D., Zhuang X., Xiang X., Liu Y., Zhang S., Liu C., Barnes S., Grizzle W., Miller D., Zhang H. G. (2010). A novel
nanoparticle drug delivery system: The anti-inflammatory activity
of curcumin is enhanced when encapsulated in exosomes. Mol. Ther..

[27] Ramezani R., Mohammadian M., Hosseini E. S., Zare M. (2023). The effect of bovine
milk lactoferrin-loaded exosomes (exoLF) on human MDA-MB-231 breast
cancer cell line. BMC Complement Med. Ther..

[28] Johnsen K. B., Gudbergsson J. M., Skov M. N., Pilgaard L., Moos T., Duroux M. (2014). A comprehensive
overview of exosomes as drug delivery
vehicles - Endogenous nanocarriers for targeted cancer therapy. Biochim Biophys Acta Rev. Cancer.

[29] Kooijmans S. A. A., Stremersch S., Braeckmans K., De Smedt S. C., Hendrix A., Wood M. J. A., Schiffelers R. M., Raemdonck K., Vader P. (2013). Electroporation-induced siRNA precipitation obscures the efficiency
of siRNA loading into extracellular vesicles. J. Controlled Release.

[30] Hood J. L., Scott M. J., Wickline S. A. (2014). Maximizing exosome
colloidal stability
following electroporation. Anal. Biochem..

[31] Palakurthi S. S., Shah B., Kapre S., Charbe N., Immanuel S., Pasham S., Thalla M., Jain A., Palakurthi S. (2024). A comprehensive
review of challenges and advances in exosome-based drug delivery systems. Nanoscale Adv..

[32] Li Y., Xing L., Wang L., Liu X., Wu L., Ni M., Zhou Z., Li L., Liu X., Huang Y. (2023). Milk-derived
exosomes as a promising vehicle for oral delivery of hydrophilic biomacromolecule
drugs. Asian J. Pharm. Sci..

[33] Izumi H., Kosaka N., Shimizu T., Sekine K., Ochiya T., Takase M. (2012). Bovine milk contains microRNA and
messenger RNA that
are stable under degradative conditions. J.
Dairy Sci..

[34] Zhou Q., Li M., Wang X., Li Q., Wang T., Zhu Q., Zhou X., Wang X., Gao X., Li X. (2012). Immune-related
microRNAs are abundant in breast milk exosomes. Int. J. Biol. Sci..

[35] Komine-Aizawa S., Ito S., Aizawa S., Namiki T., Hayakawa S. (2020). Cow milk exosomes activate
NK cells and γδT cells in human PBMCs in vitro. Immunol. Med..

[36] Samuel M., Chisanga D., Liem M., Keerthikumar S., Anand S., Ang C. S., Adda C. G., Versteegen E., Jois M., Mathivanan S. (2017). Bovine milk-derived exosomes from
colostrum are enriched with proteins implicated in immune response
and growth. Sci. Rep..

[37] Bradford M. M. (1976). A Rapid
and Sensitive Method for the Quantitation of Microgram Quantities
of Protein Utilizing the Principle of Protein-Dye Binding. Anal. Biochem..

[38] Tian Y., Li S., Song J., Ji T., Zhu M., Anderson G. J., Wei J., Nie G. (2014). A doxorubicin
delivery platform using engineered natural
membrane vesicle exosomes for targeted tumor therapy. Biomaterials.

